# Antibiotic use and risk of autism spectrum disorder and attention-deficit/hyperactivity disorder: a population-based cohort study

**DOI:** 10.1186/s13034-024-00774-4

**Published:** 2024-07-11

**Authors:** Kai-Lin Yang, Ting-An Yen, Fang-Ju Lin, Chien-Ning Hsu, Chi-Chuan Wang

**Affiliations:** 1 Graduate Institute of Clinical Pharmacy, College of Medicine, National Taiwan University, Taipei, Taiwan; 2 Department of Pediatrics, National Taiwan University College of Medicine and Hospital, Taipei, Taiwan; 3 Department of Pharmacy, National Taiwan University Hospital, Taipei, Taiwan; 4 School of Pharmacy, College of Medicine, National Taiwan University, Taipei, Taiwan; 5 Department of Pharmacy, Kaohsiung Chang Gung Memorial Hospital, Kaohsiung, Taiwan; 6 School of Pharmacy, Kaohsiung Medical University, Kaohsiung, Taiwan

**Keywords:** Gut microbiota, Gut–brain axis, Antibacterial agents, Autism spectrum disorder, Attention-deficit/hyperactivity disorder

## Abstract

**Background:**

The gut microbiota is believed to influence neurodevelopment through the gut–brain axis, but prior studies have shown inconsistent results regarding early childhood antibiotic exposure and subsequent risk of autism spectrum disorder (ASD) and attention-deficit/hyperactivity disorder (ADHD). The purpose of this study was to evaluate the hypothesis that exposure to antibacterial agents in the first 2 years of life increases the risk of ASD and/or ADHD.

**Methods:**

This was a retrospective cohort study using 2003–2019 data from the National Health Insurance Research Database in Taiwan. Livebirths born between 2004 and 2016 were identified and separated into singleton, full sibling, and exposure-discordant sibling pair cohorts. The exposure group included children who filled at least one prescription for antibacterial agents between 0 and 2 years old in outpatient settings. The outcome, ASD and/or ADHD, was defined by at least one inpatient or outpatient diagnosis. The maximum follow-up age was 15 years in this study. Potential neonatal, maternal and paternal confounders were adjusted for. Cox proportional hazards models were used to estimate the relative event risk.

**Results:**

The final sample contained 946,581 children in the singleton cohort, 1,142,693 children in the full sibling cohort, and 352,612 children in the exposure-discordant sibling pair cohort. Antibiotic exposure marginally increased the risk of ASD and/or ADHD in the singleton cohort (adjusted hazard ratio [aHR]: 1.06, 95% confidence interval [CI]: 1.04–1.07) and in the full sibling cohort (aHR: 1.03, 95% CI: 1.01–1.04). A slight decrease in the risk of ASD and/or ADHD was observed in the exposure-discordant sibling pair cohort (aHR: 0.92, 95% CI: 0.90–0.94).

**Conclusions:**

The results suggest that early life antibiotic exposure has minimal impact on the risk of ASD and/or ADHD. Given that the estimated effects are marginal and close to null, concerns about ASD and/or ADHD risk increase should not postpone or deter timely and reasonable antibiotic use.

**Supplementary Information:**

The online version contains supplementary material available at 10.1186/s13034-024-00774-4.

## Background

The gut microbiota (GM) is believed to influence neurodevelopment through the gut–brain axis (GBA) [1]. Previous studies have shown that compared with typically developing children, children with autism spectrum disorder (ASD) and/or attention-deficit/hyperactivity disorder (ADHD) have different GM compositions [2–4]. Several studies have shown that prenatal and postnatal antibiotic exposure in childhood is associated with a 10–50% increase in the risk of subsequent ASD and ADHD [5–9], whereas others have shown a null association between early childhood antibiotic exposure and subsequent ASD and ADHD [10–12]. These inconsistent findings may be due to the heterogeneity of the study designs such as the age and composition of the cohorts (e.g., sibling or nonsibling cohorts), or confounding adjustment.

The exact interplay of the GBA during the postnatal period of growth is not well understood [13]. The GM starts at birth and is gradually established until 2 to 3 years of age [14, 15]. Among the different phases of GM formation, the first 3 to 14 months of life are significantly influenced by dietary factors (e.g., breast milk and solid food) [16]. Given that the critical period of GM development occurs in early childhood, the purpose of this study was to evaluate the correlation between exposure to antibacterial agents in early childhood and the risk of ASD and/or ADHD in an Asian population. We hypothesized that children exposed to antibiotics in early childhood have an increased risk of ASD and/or ADHD later in life.

## Methods

### Study design

This was a nationwide retrospective cohort study. We used 2003–2019 deidentified claims data from the National Health Insurance Research Database (NHIRD). The NHIRD contains information on insurance enrollment, outpatient claims, inpatient claims, pharmacy claims, catastrophic illness registry, birth certificates, and causes of death. The Maternal and Child Health Database (MCHD) was used to link children and their parents [17].

### Study population

This study included all liveborn pregnancies in Taiwan between January 1, 2004 and December 31, 2016. We excluded pregnancies with a gestational age < 37 weeks, gestational size < 2500 g, missing parental ID for linkage, and missing birth date or 5-min Apgar score data. In addition to using the children’s and the parents’ IDs for matching, we also utilized the children’s birth year and sex to identify their corresponding parents. Children who could not be matched with their corresponding parents based on these three variables (ID, birth year, and sex) were excluded. Children who died or were diagnosed with ASD or ADHD before the age of 2 years were also excluded for two reasons. First, ASD/ADHD can be more reliably diagnosed around the ages of 4 to 5 years, as children aged 2 to 3 years are still in a developmental stage where it is challenging to determine whether their behaviors constitute pathological signs. Second, as we assessed antibiotic exposure during the time window between 0 and 2 years, diagnosing ASD/ADHD within the same time frame complicates establishing the temporal relationship between antibiotic exposure and the outcomes of interest.

After applying the exclusion criteria, we separated the study sample into singleton and full sibling cohorts. Next, we identified families with two children who exhibited different exposure statuses, creating exposure-discordant sibling pairs from the full sibling cohort. These steps allowed us to further adjust for potential hereditary and environmental confounders between different families.

### Exposure definition

The exposure group included children who filled at least one prescription for antibacterial agents between 0 and 2 years old in outpatient settings. We selected 0–2 years of age as the exposure window for the main analysis because the GM converges toward adult-like microbiota in the first two years of life [15]. The Anatomical Therapeutic Chemical (ATC) codes were used to classify the antibacterial agents (Table A1). We calculated cumulative exposure as days of antibiotic exposure and the total number of types of antibiotics prescribed during the first 2 years of life to determine whether there was a dose-response relationship between antibiotic exposure and the risk of ASD/ADHD.

### Outcome assessment

The outcome of interest in this study was the composite of ASD and ADHD, defined by at least one inpatient or outpatient diagnosis. The International Classification of Disease, Ninth Revision, Clinical Modification (ICD-9-CM) codes and ICD-10-CM codes were used to identify the medical conditions (Table A2). The literature generally reports good validation of this outcome definition [18].

### Follow-up

We began the follow-up on the children’s second birthday until the occurrence of the outcome of interest, death, or the end of the observation (i.e., December 31, 2019). The maximum follow-up age was 15 years.

### Covariates

Potential confounders and risk factors for ASD/ADHD were divided into three main categories: neonatal, maternal, and paternal factors. In general, neonatal covariates indicate children’s health conditions and thus could be associated with the probability of antibacterial agent exposure and the risk of ASD/ADHD. Maternal and paternal covariates were considered potential risk factors for ASD/ADHD. The neonatal covariates included sex, mode of delivery (vaginal delivery or cesarean delivery), gestational age, birth weight, first birth order at delivery (yes/no), singleton birth (yes/no), 5-min Apgar score (≥ 7 points or not), birth complications (yes/no), season of birth, year of birth, and inpatient antibiotic exposure (yes/no). Birth complications included maternal fever during childbirth, premature rupture of the membrane, placenta previa, and mass bleeding. Inpatient antibiotic exposure indicated whether a child received intravenous antibiotics in an inpatient setting at 0–2 years of age.

Maternal covariates included maternal psychiatric and neurological diseases, asthma, systemic inflammatory disorders, diabetes mellitus (type 1, type 2, and gestational), proteinuria, and hypertensive disorders during pregnancy. Psychiatric and neurological diseases included mood (affective) disorders, anxiety disorders, schizophrenia, epilepsy, ASD, and ADHD. We also controlled for prenatal exposure to antibiotics during pregnancy (inpatient only, outpatient only, both inpatient and outpatient, none) and the use of antidepressants during pregnancy (yes/no). Paternal covariates included psychiatric and neurological diseases, asthma, and systemic inflammatory disorders. The ICD-9-CM and ICD-10-CM codes used to identify maternal and paternal conditions are listed in Table A2.

### Statistical analysis

In the singleton cohort, one-to-one greedy nearest neighbor matching without replacement using the propensity score (PS) was performed to balance the baseline characteristics between the two groups, using a caliper of width equal to 0.2 times the standard deviation of the logit of the PS [19] Absolute standardized mean differences less than 0.1 were used as a threshold to define whether the balances were achieved across covariates [20]. The remaining unbalanced covariates were adjusted in the regression model.

Multivariate Cox proportional hazards regression models were used to estimate the risk of ASD and ADHD as a composite endpoint for children exposed to antibacterial agents versus their nonexposed counterparts. For the singleton cohort, the Cox model included the exposure status and all covariates that remained unbalanced after PS matching. For the full sibling cohort and the exposure-discordant sibling pair cohort, traditional covariate adjustment with robust standard errors was performed to maintain family clusters in the sibling analysis. All the statistical procedures were performed with SAS version 9.4 (SAS Institute Inc., Cary, NC, USA). This study was reviewed and approved by the National Taiwan University Hospital Research Ethics Committee (No. 202111105RINA). All analyses were conducted using deidentified patient–level records. Thus, the requirement for informed consent by individual patients was waived by the Institutional Review and Ethics Board.

### Sensitivity analysis

Several sensitivity analyses were conducted to confirm the robustness of the results. First, we shortened the exposure time window from 0–2 to 0–1 years of age and extended it from 0–2 to 0–3 years of age. Second, instead of using a group unexposed to antibacterial agents as a control, we used children exposed to antiviral agents as an active comparator to reduce potential confounding by indication when comparing drug users and nonusers. As we evaluated the class effect of antibacterial agents in this study, finding an active comparator with the same indication was challenging. Although antibacterial and antiviral agents do not share the same indication, both are used to treat infections. Using antiviral agents as the comparator helps to mitigate confounding by indication because children taking antiviral agents are likely to have infections and require medication, making them more comparable to antibiotic users than non-antibiotic users, who may not have any infections. We selected antiviral agents as active comparators due to their similar treatment purpose and the premise that GM might not be affected by antiviral agents. The ATC codes used to identify antiviral agents are provided in Table A1. Third, we adopted a stricter diagnostic algorithm to identify ASD and/or ADHD by requiring at least two outpatient diagnoses or one inpatient diagnosis. Fourth, we analyzed ASD and ADHD as two separate outcomes instead of having them as a composite outcome.

## Results

### Main analysis

**Fig. 1:**
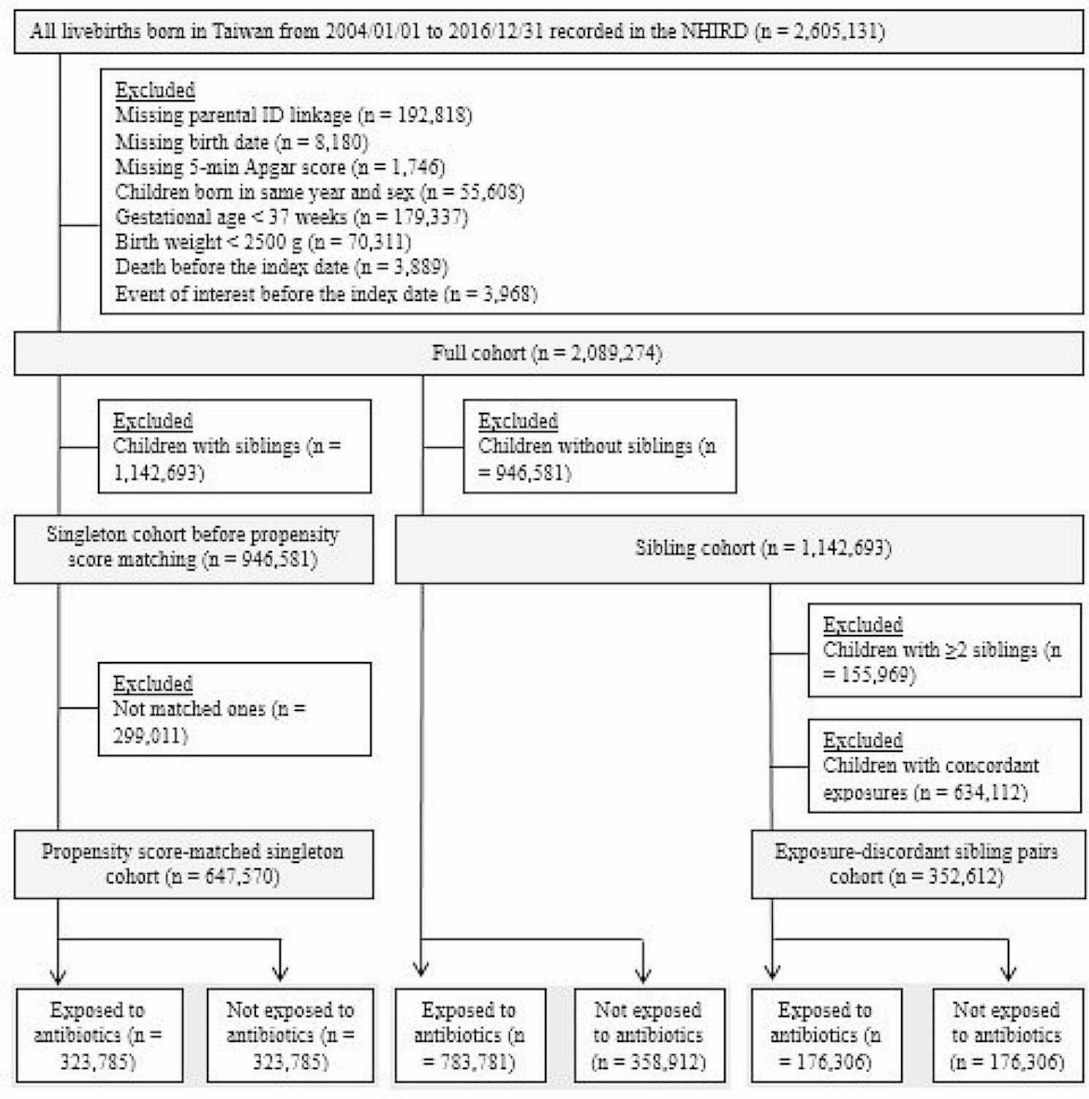
Sample selection flow chart for the study cohorts

We identified 2,617,069 livebirths from 2004 to 2016 in Taiwan. Figure 1 presents the sample selection flow chart. The final sample contained 2,089,274 children, with 946,581 in the singleton cohort and 1,142,693 in the full sibling cohort. In the singleton cohort, 619,896 children (65.49%) were exposed to antibiotics during the first 2 years of life, and 323,785 pairs of children were matched by PS. The baseline characteristics were well-balanced after PS matching. Among the full sibling cohort, 783,781 (68.59%) were exposed to antibiotics during the first 2 years of life. We identified 176,306 exposure-discordant pairs from the full sibling cohort.

The baseline characteristics of the singleton cohort before and after PS matching are shown in Table 1. Compared to the antibiotic-unexposed group, the antibiotic-exposed group had a greater proportion of males (55.24% vs. 49.99%), was less likely to be born between 2013 and 2016 (31.65% vs. 44.40%), and was more likely to be exposed to antibiotics in an inpatient setting (26.35% vs. 12.22%). We also observed a greater proportion of maternal anxiety disorders (29.65% vs. 24.44%) and a greater proportion of antibiotic exposure during pregnancy in both inpatient and outpatient settings (32.46% vs. 26.30%) in the antibiotic-exposed group than in the unexposed group. These covariates were all well-balanced after PS matching.

**Table 1:** Basic characteristics of the singleton cohort before and after propensity score matching

n (%)	Before propensity score matching (*n* = 946,581)		SMD	After propensity score matching (*n* = 647,570)		SMD
	Exposed (*n* = 619,896)	Unexposed (*n* = 326,685)		Exposed (*n* = 323,785)	Unexposed (*n* = 323,785)	
**Neonatal covariates**						
Gestational weeks (week), mean (SD)	39.40 (1.96)	39.48 (2.01)	− 0.04	39.47 (2.00)	39.47 (2.00)	− 0.01
Birth weight (g), mean (SD)	3214 (466)	3208 (458)	0.01	3208 (458)	3209 (458)	< 0.01
Male sex	342,404 (55.24)	163,308 (49.99)	0.11	163,476 (50.49)	163,180 (50.40)	− 0.06
Birth complications	96,774 (15.61)	53,337 (16.33)	− 0.02	52,695 (16.27)	52,530 (16.22)	< 0.01
Season of birth			0.03			< 0.01
Spring	147,640 (23.82)	76,065 (23.28)		75,344 (23.27)	75,550 (23.33)	
Summer	152,266 (24.56)	80,676 (24.70)		79,882 (24.67)	79,838 (24.66)	
Autumn	167,323 (26.99)	90,714 (27.77)		90,048 (27.81)	89,741 (27.72)	
Winter	152,667 (24.63)	79,230 (24.25)		78,511 (24.25)	78,656 (24.29)	
Year of birth			0.31			< 0.01
2004–2006	205,862 (33.21)	68,338 (20.92)		68,829 (21.26)	68,338 (21.11)	
2007–2009	120,566 (19.45)	57,609 (17.63)		58,050 (17.93)	57,609 (17.79)	
2010–2012	97,283 (15.69)	55,704 (17.05)		56,064 (17.32)	55,696 (17.20)	
2013–2016	196,185 (31.65)	145,034 (44.40)		140,842 (43.50)	142,142 (43.90)	
First birth order in this delivery	619,509 (99.94)	326,512 (99.95)	< − 0.01	323,599 (99.94)	323,612 (99.95)	< − 0.01
Singleton birth	618,890 (99.84)	326,192 (99.85)	< − 0.01	323,267 (99.84)	323,293 (99.85)	< − 0.01
Vaginal delivery	389,763 (62.88)	212,195 (64.95)	− 0.04	209,609 (64.74)	209,790 (64.79)	< − 0.01
Normal 5-min Apgar score	619,016 (99.86)	326,269 (99.87)	< − 0.01	323,377 (99.87)	323,379 (99.87)	< − 0.01
Inpatient antibiotic exposure	163,325 (26.35)	39,922 (12.22)	0.36	40,426 (12.49)	39,922 (12.33)	0.01
**Maternal covariates**						
Mood (affective) disorders	50,121 (8.09)	21,672 (6.63)	0.06	21,868 (6.75)	21,630 (6.68)	< 0.01
Anxiety disorders	183,821 (29.65)	79,843 (24.44)	0.12	80,268 (24.79)	79,795 (24.64)	< 0.01
Schizophrenia	3305 (0.53)	1518 (0.46)	0.01	1519 (0.47)	1508 (0.47)	< 0.01
Epilepsy	6203 (1.00)	2809 (0.86)	0.02	2828 (0.87)	2795 (0.86)	< 0.01
ASD	58 (0.01)	32 (0.01)	< 0.01	28 (0.01)	31 (0.01)	< − 0.01
ADHD	1,070 (0.17)	501 (0.15)	0.01	524 (0.16)	499 (0.15)	< 0.01
Asthma	113,470 (18.30)	48,646 (14.89)	0.09	49,124 (15.17)	48,626 (15.02)	< 0.01
Systemic inflammatory disorders	34,195 (5.52)	15,932 (4.88)	0.03	15,909 (4.91)	15,879 (4.90)	< 0.01
Diabetes	49,930 (8.05)	24,970 (7.64)	0.02	24,982 (7.72)	24,891 (7.69)	< 0.01
Proteinuria and hypertensive disorders	18,808 (3.03)	9438 (2.89)	0.01	9482 (2.93)	9399 (2.90)	< 0.01
Antibiotic exposure during pregnancy			0.16			< 0.01
Unexposed	88,556 (14.29)	59,822 (18.31)		57,589 (17.79)	58,038 (17.92)	
Inpatient only	286,307 (46.19)	160,339 (49.08)		159,154 (49.15)	159,224 (49.18)	
Outpatient only	43,818 (7.07)	20,592 (6.30)		20,728 (6.40)	20,592 (6.36)	
Both inpatient and outpatient	201,215 (32.46)	85,932 (26.30)		86,314 (26.66)	85,931 (26.54)	
Antidepressant exposure during pregnancy	4282 (0.69)	1895 (0.58)	0.01	1913 (0.59)	1892 (0.58)	< 0.01
**Paternal covariates**						
Mood (affective) disorders	30,658 (4.95)	14,339 (4.39)	0.03	14,373 (4.44)	14,275 (4.41)	< 0.01
Anxiety disorders	129,130 (20.83)	59,877 (18.33)	0.06	60,194 (18.59)	59,702 (18.44)	< 0.01
Schizophrenia	3464 (0.56)	1533 (0.47)	0.01	1557 (0.48)	1531 (0.47)	< 0.01
Epilepsy	7539 (1.22)	3447 (1.06)	0.02	3473 (1.07)	3439 (1.06)	< 0.01
ASD	85 (0.01)	32 (0.01)	< 0.01	26 (0.01)	32 (0.01)	< − 0.01
ADHD	1,281 (0.21)	687 (0.21)	< 0.01	706 (0.22)	687 (0.21)	< 0.01
Asthma	88,944 (14.35)	40,731 (12.47)	0.06	40,974 (12.65)	40,668 (12.56)	< 0.01
Systemic inflammatory disorders	16,086 (2.59)	7730 (2.37)	0.02	7772 (2.40)	7704 (2.38)	< 0.01

SD, standard deviation; SMD, standardized mean differences; ASD, autism spectrum disorder; ADHD, attention-deficit/hyperactivity disorder

The baseline characteristics of the full sibling cohort and exposure-discordant sibling pair cohort are presented in Table 2. Within the full sibling cohort, a lower proportion of children in the exposed group than in the unexposed group, were born from 2013 to 2016 (26.7% vs. 33.31%) and had prenatal exposure to antibiotics (16.54% vs. 19.76%), but a greater proportion of the children in the exposed group had neonatal inpatient antibiotic exposure (26.40% vs. 12.15%). In the exposure-discordant sibling pair cohort, a greater proportion of children in the exposed group were male (53.61% vs. 47.78%) and had neonatal inpatient exposure to antibiotics (23.23% vs. 13.07%).

**Table 2:** Basic characteristics of the sibling cohort and the exposure-discordant sibling pairs cohort

n (%)	Full sibling cohort (*n* = 1,142,693)		SMD	Exposure-discordant sibling pairs cohort (*n* = 352,612)		SMD
	Exposed (*n* = 783,781)	Unexposed (*n* = 358,912)		Exposed (*n* = 176,306)	Unexposed (*n* = 176,306)	
**Neonatal covariates**						
Gestational weeks (week), mean (SD)	39.40 (1.96)	39.45 (1.99)	− 0.03	39.39 (1.95)	39.45 (1.99)	− 0.03
Birth weight (g), mean (SD)	3,213 (463)	3,206 (456)	0.02	3,209 (462)	3,204 (456)	0.01
Male sex	411,755 (52.53)	171,442 (47.77)	0.10	94,516 (53.61)	84,239 (47.78)	0.12
Birth complications	107,082 (13.66)	50,126 (13.97)	− 0.01	24,191 (13.72)	25,237 (14.31)	− 0.02
Season of birth			0.03			0.03
Spring	188,764 (24.08)	85,930 (23.94)		42,275 (23.98)	42,263 (23.97)	
Summer	193,851 (24.73)	89,024 (24.80)		42,945 (24.36)	43,913 (24.91)	
Autumn	213,414 (27.23)	98,824 (27.53)		48,896 (27.73)	48,211 (27.35)	
Winter	187,752 (23.95)	85,134 (23.72)		42,190 (23.93)	41,919 (23.78)	
Year of birth			0.16			0.03
2004–2006	158,717 (20.25)	57,245 (15.95)		30,030 (17.03)	30,894 (17.52)	
2007–2009	211,791 (27.02)	86,398 (24.07)		43,316 (24.57)	44,196 (25.07)	
2010–2012	203,995 (26.03)	95,720 (26.67)		46,854 (26.58)	46,500 (26.37)	
2013–2016	209,278 (26.70)	119,549 (33.31)		56,106 (31.82)	54,716 (31.03)	
First birth order in this delivery	782,721 (99.86)	358,355 (99.84)	0.01	176,134 (99.90)	176,139 (99.91)	< 0.01
Singleton birth	781,607 (99.72)	357,753 (99.68)	0.01	175,954 (99.80)	175,946 (99.80)	< 0.01
Vaginal delivery	534,332 (68.17)	252,527 (70.36)	− 0.05	121,325 (68.82)	121,718 (69.04)	− 0.01
Normal 5-min Apgar score	783,080 (99.91)	358,579 (99.91)	< 0.01	176,152 (99.91)	176,129 (99.90)	< 0.01
Inpatient antibiotic exposure	206,916 (26.40)	43,590 (12.15)	0.37	40,952 (23.23)	23,047 (13.07)	0.27
**Maternal covariates**						
Mood (affective) disorders	49,471 (6.31)	19,214 (5.35)	0.04	10,142 (5.75)	10,142 (5.75)	< 0.01
< Anxiety disorders	203,919 (26.02)	78,638 (21.91)	0.10	41,506 (23.54)	41,506 (23.54)	< 0.01
Schizophrenia	2,423 (0.31)	1,014 (0.28)	0.01	509 (0.29)	509 (0.29)	< 0.01
Epilepsy	6,358 (0.81)	2,621 (0.73)	0.01	1,304 (0.74)	1,304 (0.74)	< 0.01
ASD	61 (0.01)	24 (0.01)	0.01	9 (0.01)	9 (0.01)	< 0.01
ADHD	950 (0.12)	427 (0.12)	0.01	212 (0.12)	212 (0.12)	< 0.01
Asthma	145,271 (18.53)	55,961 (15.59)	0.08	29,344 (16.64)	29,344 (16.64)	< 0.01
Systemic inflammatory disorders	40,635 (5.18)	16,893 (4.71)	0.02	8,754 (4.97)	8,754 (4.97)	< 0.01
Diabetes	59,306 (7.57)	25,318 (7.05)	0.02	12,766 (7.24)	12,921 (7.33)	< − 0.01
Proteinuria and hypertensive disorders	16,915 (2.16)	7,111 (1.98)	0.01	3,491 (1.98)	3,719 (2.11)	− 0.01
Antibiotic exposure during pregnancy			0.13			0.02
Unexposed	129,662 (16.54)	70,908 (19.76)		32,603 (18.49)	32,273 (18.31)	
Inpatient only	344,700 (43.98)	169,497 (47.23)		81,591 (46.28)	82,534 (46.81)	
Outpatient only	66,111 (8.43)	26,233 (7.31)		13,627 (7.73)	13,414 (7.61)	
Both inpatient and outpatient	243,308 (31.04)	92,274 (25.71)		48,485 (27.50)	48,085 (27.27)	
Antidepressant exposure during pregnancy	3,064 (0.39)	1,274 (0.35)	0.01	636 (0.36)	680 (0.39)	< − 0.01
**Paternal covariates**						
Mood (affective) disorders	26,711 (3.41)	11,092 (3.09)	0.02	6,439 (3.65)	6,439 (3.65)	< 0.01
Anxiety disorders	151,987 (19.39)	62,560 (17.43)	0.05	32,139 (18.23)	32,139 (18.23)	< 0.01
Schizophrenia	2,607 (0.33)	1,115 (0.31)	< 0.01	519 (0.29)	519 (0.29)	< 0.01
Epilepsy	7,386 (0.94)	3,127 (0.87)	0.01	1,549 (0.88)	1,549 (0.88)	< 0.01
ASD	79 (0.01)	28 (0.01)	< 0.01	17 (0.01)	17 (0.01)	< 0.01
ADHD	1,217 (0.16)	557 (0.16)	< 0.01	266 (0.15)	266 (0.15)	< 0.01
Asthma	121,000 (15.44)	49,262 (13.73)	0.05	25,397 (14.41)	25,397 (14.41)	< 0.01
Systemic inflammatory disorders	20,793 (2.65)	8,958 (2.50)	0.01	4,567 (2.59)	4,567 (2.59)	< 0.01

SD, standard deviation; SMD, standardized mean differences; ASD, autism spectrum disorder; ADHD, attention-deficit/hyperactivity disorder

Table 3 shows the effect estimates of early childhood antibiotic exposure and the risk of ASD and/or ADHD in the singleton, sibling, and exposure-discordant sibling pair cohorts. Antibiotic exposure slightly increased the risk of ASD and/or ADHD in the singleton cohort after PS matching (adjusted hazard ratio [aHR]: 1.06, 95% confidence interval [CI]: 1.04–1.07), and similar findings were observed in the full sibling cohort (aHR: 1.03, 95% CI: 1.01–1.04). However, in the exposure-discordant sibling cohort, the risk of ASD and/or ADHD was slightly lower among children who were exposed to antibiotics at 0–2 years of age than among those who were not exposed (aHR: 0.92, 95% CI: 0.90–0.94).

**Table 3:** Associations between antibiotic exposure in 0–2 years old and subsequent autism spectrum disorder and/or attention-deficit/hyperactivity disorder

Study cohorts	Number of subjects	Number of events	Median follow-up time (years)	Incidence rate (1,000 person-years)	Unadjusted hazard ratio (95% CI)	Adjusted hazard ratio ^a^ (95% CI)
Singleton cohort						
Exposed group	619,896	57,067	7.37	12.31 (12.21–12.41)	1.10 (1.08–1.11)	1.06 (1.04–1.07)
Unexposed group	326,685	23,821	5.28	11.60 (11.46–11.75)	*Reference*	*Reference*
Full sibling cohort						
Exposed group	783,781	65,985	6.97	11.71 (11.62–11.80)	1.12 (1.10–1.14)	1.03 (1.01–1.04)
Unexposed group	358,912	25,385	6.25	10.56 (10.44–10.69)	*Reference*	*Reference*
Exposure-discordant sibling pairs cohort						
Exposed group	176,306	13,288	6.43	11.12 (10.94–11.31)	0.99 (0.97–1.02)	0.92 (0.90–0.94)
Unexposed group	176,306	13,641	6.44	11.25 (11.06–11.44)	*Reference*	*Reference*

^a^Baseline covariates were balanced in the singleton cohort by propensity score matching for the singleton cohort and were adjusted by multivariate Cox regression models in the full sibling cohort and exposure-discordant sibling pairs cohort

Similar results were found in the analyses with exposure classified by the cumulative number of days and the cumulative number of classes of antibiotic used (Table 4). A slightly greater risk of ASD and/or ADHD was observed in the singleton and full sibling cohorts, while a slightly lower risk of ASD and/or ADHD was observed in the exposure-discordant sibling pair cohort. Notably, the risk did not increase with increasing duration or number of antibiotic classes used.

**Table 4:** Associations between antibiotics exposure in 0–2 years old and subsequent autism spectrum disorder and/or attention-deficit/hyperactivity disorder, by cumulative days and cumulative classes of antibiotic exposure

Study cohorts	Number of subjects	Number of events	Median follow-up time (years)	Incidence rate (1,000 person-years)	Adjusted hazard ratio^a^ (95% CI)
**Singleton cohort**					
*Cumulative days*					
0	323,785	23,779	5.34	11.63 (11.48–11.77)	*Reference*
1–3	80,039	5,676	4.69	12.15 (11.84–12.47)	1.03 (1.00–1.06)
4–7	66,444	5,044	4.94	12.57 (12.23–12.91)	1.07 (1.04–1.11)
8–14	74,457	5,766	5.37	12.22 (11.91–12.54)	1.05 (1.02–1.08)
≥ 15	102,845	8,730	6.49	12.07 (11.82–12.32)	1.06 (1.04–1.09)
*Cumulative categories*					
0	323,785	23,779	5.34	11.63 (11.48–11.77)	*Reference*
1	186,987	13,693	4.80	12.39 (12.19–12.60)	1.05 (1.03–1.08)
2	101,040	8,213	5.92	12.15 (11.90-12.42)	1.06 (1.03–1.08)
3	29,911	2,706	7.83	11.76 (11.33–12.21)	1.05 (1.01–1.09)
≥ 4	5,847	604	10.13	11.46 (10.58–12.40)	1.06 (0.98–1.15)
**Full sibling cohort**					
*Cumulative days*					
0	358,912	25,385	6.25	10.56 (10.44–10.69)	*Reference*
1–3	161,397	12,625	6.71	11.12 (10.93–11.31)	1.02 (1.00–1.05)
4–7	145,755	11,783	6.85	11.35 (11.15–11.56)	1.02 (1.00–1.05)
8–14	178,326	14,805	6.97	11.52 (11.33–11.70)	1.02 (1.00–1.04)
≥ 15	298,303	26,772	7.18	12.31 (12.16–12.45)	1.04 (1.02–1.06)
*Cumulative categories*					
0	358,912	25,385	6.25	10.56 (10.44–10.69)	*Reference*
1	407,749	32,228	6.61	11.39 (11.27–11.51)	1.02 (1.01–1.04)
2	266,520	23,299	7.17	11.96 (11.81–12.11)	1.04 (1.02–1.06)
3	91,425	8,590	7.85	12.18 (11.93–12.44)	1.02 (1.00–1.05)
≥ 4	18,087	1,868	8.63	12.34 (11.80-12.91)	1.04 (0.99–1.09)
**Exposure-discordant pair sibling cohort**					
*Cumulative days*					
0	176,306	13,641	6.44	11.25 (11.06–11.44)	*Reference*
1–3	48,422	3,533	6.28	10.88 (10.53–11.24)	0.94 (0.91–0.98)
4–7	39,154	2,865	6.36	10.85 (10.46–11.25)	0.91 (0.87–0.95)
8–14	40,739	3,061	6.46	11.03 (10.65–11.42)	0.90 (0.87–0.94)
≥ 15	47,991	3,829	6.59	11.67 (11.31–12.05)	0.92 (0.89–0.95)
*Cumulative categories*					
0	176,306	13,641	6.44	11.25 (11.06–11.44)	*Reference*
1	108,726	7,844	6.21	10.91 (10.67–11.15)	0.92 (0.89–0.95)
2	52,047	4,042	6.68	11.19 (10.85–11.54)	0.90 (0.87–0.94)
3	13,400	1,215	7.14	12.46 (11.78–13.17)	0.98 (0.92–1.04)
≥ 4	2,133	187	8.08	11.03 (9.56–12.72)	0.87 (0.75–1.00)

^a^Baseline covariates were balanced in the singleton cohort by propensity score matching for the singleton cohort and were adjusted by multivariate Cox regression models in the full sibling cohort and exposure-discordant sibling pairs cohort

Cumulative days were calculated as cumulative days of antibiotic exposure during the first 2 years of life; cumulative categories were calculated as the number of antibiotic classes prescribed during the first 2 years of life

### Sensitivity analyses

Table A3 summarizes the results of the first three sensitivity analyses. When the exposure window was decreased to 0–1 year of age, the risk of ASD and/or ADHD decreased in all three cohorts. A null association between antibiotic exposure and the risk of ASD and/or ADHD was observed in both the singleton and full sibling cohorts (aHR_singleton_: 0.99, 95% CI: 0.97–1.00; aHR_sibling_: 0.99, 95% CI: 0.98–1.00). When we extended the exposure window to 0–3 years, the results were consistent with the main findings across all three cohorts. The aHR was 1.14 (95% CI: 1.11–1.17) in the singleton cohort, 1.09 (95% CI: 1.07–1.11) in the full sibling cohort, and 0.97 (95% CI: 0.94–1.00) in the exposure-discordant sibling pair cohort.

In the sensitivity analysis that utilized antiviral agents as an active comparator, consistent results were found across the three cohorts compared with the main analysis. However, due to the relatively small sample sizes, all the confidence intervals became slightly wider. The results remained robust across the three cohorts with the stricter outcome definition compared with the results from the main analysis.

The results of the sensitivity analysis that evaluated ASD and ADHD as two individual outcomes are presented in Table A4. We found that antibiotic exposure at 0–2 year of age was associated with a small decrease in the risk of ASD (aHR_Singletons_: 0.91, 95% CI: 0.88–0.95; aHR_Exposure−discordant pairs siblings_: 0.88, 95% CI: 0.83–0.93). However, no differences were found regarding the risk of ADHD in either the singleton cohort (aHR: 1.08, 95% CI: 1.06–1.10) or the exposure-discordant pairs sibling cohort (aHR: 0.92, 95% CI: 0.90–0.94).

## Discussion

In our study, antibiotic exposure during the first 2 years of life was associated with a slight increase in the risk of subsequent ASD and/or ADHD in children in the PS-matched singleton cohort and in the full sibling cohort, while a slight decrease in the risk was observed in the exposure-discordant sibling pair cohort. Despite conducting several sensitivity analyses to ensure the robustness of our results, we cannot completely rule out the possibility of unmeasured confounding influencing our study findings. Given that all of the effect estimates were close to one, our results suggest that the impact of antibiotic use during children’s first 2 years of life on the risk of ASD and/or ADHD is minimal. Therefore, concerns about the increased risk of ASD and/or ADHD should not delay or discourage the timely and appropriate use of antibiotics.

Similar to our findings, the effect estimates from previous studies were also close to null. A prior study showed a marginal protective effect of antibiotic exposure during the first year of life on the risk of ASD in the full cohort (aHR: 0.91, 95% CI: 0.84–0.99) and a null association in the exposure-discordant sibling pair cohort (aHR: 1.03, 95% CI: 0.86–1.23) [10]. The same authors also reported no associations between first-year antibiotic exposure and the risk of ADHD in either the full cohort (aHR: 1.02, 95% CI: 0.97–1.08) or the exposure-discordant sibling pair cohort (aHR: 0.96, 95% CI: 0.98–1.03) [11]. Although we evaluated the composite risk of ASD and/or ADHD in the main analysis of this study, similar results were found when we evaluated ASD and ADHD as separate outcomes. Our results are in line with prior evidence except that we had relatively narrower confidence intervals due to large sample sizes. Together, these results suggest a marginal risk of ASD and/or ADHD related to early childhood antibiotic exposure.

An interesting finding in our study was that a slightly greater risk of ASD and/or ADHD was observed in the singleton and full sibling cohorts, but a slightly lower risk of ASD and/or ADHD was observed in the exposure-discordant sibling pair cohort. A possible explanation for this phenomenon is that parents’ within-family perceptions and behaviors of their children (e.g., parenting experience and styles, attitudes toward disease, and medication usage) are time-dependent confounders that vary between siblings [21]. Our exploratory analysis suggested that in the exposure-discordant sibling pair cohort, younger siblings were more likely to be prescribed antibiotics while older siblings were more likely to be diagnosed with ASD and/or ADHD, and the adjusted result moved closer to the null after we adjusted the birth order in the exposure-discordant sibling pair cohort. Thus, within-family parenting styles and behavior may have been unmeasured factors that affected our study results. Further studies are needed to explore this issue.

To the best of our knowledge, this is the largest retrospective cohort study to date to examine the association between antibiotic exposure in early life and subsequent ASD and/or ADHD. By virtue of the coverage of the NHIRD, the comparatively larger sample sizes provided precision to our study. In addition, we collected paternal covariates by linking the MCHD to further adjust for hereditary and behavioral factors from fathers, which made our results more robust than those of previous studies. Several sensitivity analyses were performed to ensure the validity of our results. All of the results from the sensitivity analyses were consistent with the main findings, which again supports the robustness of our study.

This study had several limitations. First, the exclusion of preterm neonates limits our generalizability, but this criterion increases the internal validity by eliminating the potential effect of preterm birth on ASD/ADHD. Second, diet information (e.g., breast feeding, prebiotics, probiotics, and other nutritional supplements) plays a vital role in growth and GM composition in early life, but these covariates were not available in the database. Parenting styles, social and economic status, and living environment were also not available for adjustment in our analysis. Third, due to the restricted sample selection criteria, the results from the singleton and exposure-discordant sibling cohorts may not be generalizable to the full cohort and the full sibling cohort, respectively. However, the results from the three study cohorts (singleton, full sibling, and exposure-discordant sibling cohorts) were consistent, and they provided estimates in different populations that complement one another. Fourth, while the sibling analysis accounted for fixed family effects, bias due to unmeasured nonshared confounding may exist as upbringing and parents’ attitudes toward disease and medication usage may change over time [22]. Last, in the NHIRD, we could only observe prescribing records without knowing the actual medication use behavior.

## Conclusions

Based on our findings, we conclude that early-life antibiotic exposure does not appear to be associated with subsequent ASD and/or ADHD. Thus, concerns about increased ASD and/or ADHD risk should not lead to the postponement or deferment of timely and reasonable antibiotic use.

### Electronic supplementary material

Below is the link to the electronic supplementary material.

Supplementary Material 1

## Data Availability

The raw data of the National Health Insurance Research Database (NHIRD) and the Maternal and Child Health Database (MCHD) are protected and are not available publicly. Access may be obtained upon reasonable request and application to the Health and Welfare Data Science Center, Ministry of Health and Welfare of Taiwan (https://dep.mohw.gov.tw/DOS/cp-5119-59201-113.html).

## Abbreviations

ASD: Autism spectrum disorder
ADHD: Attention-deficit/hyperactivity disorder
aHR: Adjusted hazard ratio
CI: Confidence interval
GM: Gut microbiota
GBA: Gut–brain axis
MCHD: Maternal and Child Health Database
NHIRD: National Health Insurance Research Database
ICD-9-CM: International Classification of Disease, Ninth Revision, Clinical Modification
ICD-10-CM: International Classification of Disease, Tenth Revision, Clinical Modification

## Declarations

### Data sharing Statement

The data that support the findings of this study are not available; access may be obtained upon reasonable request and application to the Health and Welfare Data Science Center, Ministry of Health and Welfare of Taiwan.
