# Supplementary material for: Antibiotic use and risk of autism spectrum disorder and attention-deficit/hyperactivity disorder: a population-based cohort study
Source: Child Adolesc Psychiatry Ment Health. 2024 Jul 11;18:82. doi: 10.1186/s13034-024-00774-4 (PMC11241894; doi:10.1186/s13034-024-00774-4)
Supplement: Supplementary file 1 — Supplementary Material 1 [file 13034_2024_774_MOESM1_ESM.docx]

#### **Appendix**

#### **Table A1. Anatomical Therapeutic Chemical Codes for Medications**

| **Medications** | **ATC codes** |
| --- | --- |
| Antibiotics |  |
| Beta-lactam antibiotics, penicillin | J01C |
| Other beta-lactam antibiotics | J01D |
| Sulfonamide and trimethoprim | J01E |
| Macrolide, lincosamide, and streptogramin antibiotics | J01F |
| Quinolone antibiotics | J01M |
| Tetracyclines | J01A |
| Amphenicols | J01B |
| Aminoglycosides | J01G |
| Other antibiotics or antibiotic combinations | J01R, J01X |
| Antivirals | ATC codes |
| Thiosemicarbazones | J05AB |
| Protease inhibitors | J05AE |
| Nucleoside and nucleotide reverse transcriptase inhibitors | J05AF |
| Non-nucleoside reverse transcriptase inhibitors | J05AG |
| Neuraminidase inhibitors | J05AH |
| Integrase inhibitors | J05AJ |
| Antivirals for treatment of HCV infections | J05AP |
| Antivirals for treatment of HIV infections, combinations | J05AR |
| Other antivirals | J05AX |

####

#### **Table A2. Diagnosis Codes for Study Outcomes and Baseline Covariates**

| Diseases | ICD-9-CM codes | ICD-10-CM codes |
| --- | --- | --- |
| Study Outcomes |  |  |
| Autism spectrum disorder | 299.0 | F84.0 |
|  | 299.1 | F84.3 |
|  | 299.8 | F84.5, F84.8 |
|  | 299.9 | F84.9 |
| Attention-deficit/hyperactivity disorder | 314.0 | F90.1, F90.2, F90.8, F90.9, R41.840 |
|  | 314.1, 314.2, 314.8 | F90.8 |
|  | 314.9 | F90.9 |
| Baseline Covariates |  |  |
| Maternal and paternal factors |  |  |
| Mood (affective) disorders, depression included | 296 | F30, F31, F32, F33, F34 |
| Anxiety disorder | 300.0 | F41 |
| Schizophrenia | 295 | F20, F25 |
| Epilepsy | 345 | G40 |
| Autism spectrum disorder | 299.0 | F84.0 |
|  | 299.1 | F84.3 |
|  | 299.8 | F84.5, F84.8 |
|  | 299.9 | F84.9 |
| Attention-deficit/hyperactivity disorder | 314.0 | F90.1, F90.2, F90.8, F90.9, R41.840 |
|  | 314.1, 314.2, 314.8 | F90.8 |
|  | 314.9 | F90.9 |
| Asthma | 493.x (x = 0,1,9) | J45 |
| Systemic inflammatory disorders |  |  |
| Polyarteritis nodosa and related conditions and other necrotizing vasculopathies | 446 | M30, M31 |
| Systemic lupus erythematosus, polymyalgia rheumatic, systemic sclerosis, sicca syndrome and dermatomyositis | 710, 725 | M32, M33, M34, M35.x (x = 0,1,3,5,8,9),  M36.x (x = 0,8) |
| Behçet disease | 136.1, 711.2 | M35.2 |
| Panniculitis | 729.3 | M35.6, M79.3, M79.4 |
| Hypermobility syndrome | 728.5 | M35.7 |
| Maternal only |  |  |
| Diabetes mellitus (type 1, type 2, and gestational) | 250 | E10, E11 |
|  | 648.0 | O24 |
| Proteinuria and hypertensive disorders in pregnancy, childbirth, and the puerperium | 642, 646.1, 646.2 | O10, O11, O12.2, O13, O14, O15, O16 |

ICD-9-CM codes, International Classification of Diseases, Ninth Revision, Clinical Modification codes

ICD-10-CM codes, International Classification of Diseases, Tenth Revision, Clinical Modification codes

#### **Table A3. Results of the Sensitivity Analyses**

| Studied cohorts | Number of subjects | Number of events | Median follow-up time (years) | Incidence rate  (1,000 person-years) | Adjusted  hazard ratio^a^  (95% CI) | |
| --- | --- | --- | --- | --- | --- | --- |
| Antibiotics exposure in 0–1 years of age | |  |  |  |  | |
| Singleton cohort |  |  |  |  |  | |
| Exposed group | 341,988 | 31,615 | 8.84 | 10.64 (10.53-10.76) | 0.99 (0.97–1.00) | |
| Unexposed group | 341,988 | 31,890 | 8.85 | 10.82 (10.70-10.94) | *Reference* | |
| Full sibling cohort |  |  |  |  |  | |
| Exposed group | 474,887 | 41,367 | 8.09 | 10.52 (10.42-10.62) | 0.99 (0.98–1.00) | |
| Unexposed group | 671,659 | 52,183 | 7.47 | 9.92 (9.84-10.01) | *Reference* | |
| Exposure-discordant pair sibling cohort | |  |  |  |  | |
| Exposed group | 205,512 | 17,084 | 7.67 | 10.40 (10.24-10.55) | 0.92 (0.90–0.94) | |
| Unexposed group | 205,512 | 17,883 | 7.94 | 10.65 (10.49-10.81) | *Reference* | |
| Antibiotics exposure in 0–3 years of age | |  |  |  |  | |
| Singleton cohort |  |  |  |  |  | |
| Exposed group | 209,142 | 14,245 | 4.27 | 12.84 (12.63-13.05) | 1.14 (1.11–1.17) | |
| Unexposed group | 209,142 | 12,505 | 4.27 | 11.30 (11.11-11.50) | *Reference* | |
| Full sibling cohort |  |  |  |  |  | |
| Exposed group | 908,890 | 70,063 | 5.96 | 12.44 (12.35-12.53) | 1.09 (1.07–1.11) | |
| Unexposed group | 220,306 | 12,967 | 5.11 | 10.48 (10.31-10.66) | *Reference* | |
| Exposure-discordant sibling pair cohort | |  |  |  |  | |
| Exposed group | 125,179 | 8,365 | 5.39 | 11.62 (11.37-11.87) | 0.97 (0.94–1.00) | |
| Unexposed group | 125,179 | 7,983 | 5.20 | 11.14 (10.90-11.38) | *Reference* | |
| Antivirals as an active comparator |  |  |  |  |  |  |
| Singleton cohort |  |  |  |  |  |  |
| Exposed group | 7,548 | 601 | 5.85 | 12.13 (11.21-13.14) | 1.09 (0.97–1.22) |  |
| Active comparator group | 7,548 | 550 | 5.80 | 11.16 (10.27-12.12) | *Reference* |  |
| Full sibling cohort |  |  |  |  |  |  |
| Exposed group | 559,210 | 48,534 | 7.12 | 11.89 (11.78-11.99) | 1.10 (1.00–1.22) |  |
| Active comparator group | 5,513 | 382 | 6.71 | 10.11 (9.15-11.17) | *Reference* |  |
| Exposure-discordant sibling pairs cohort | |  |  |  |  |  |
| Exposed group | 4,051 | 292 | 6.57 | 10.37 (9.26-11.63) | 0.97 (0.82–1.15) |  |
| Active comparator group | 4,051 | 286 | 6.59 | 10.36 (9.23-11.63) | *Reference* |  |
| Stricter outcome definition |  |  |  |  |  |  |
| Singleton cohort |  |  |  |  |  |  |
| Exposed group | 323,785 | 20,801 | 5.47 | 10.00 (9.87-10.14) | 1.06 (1.04–1.08) |  |
| Unexposed group | 323,785 | 19,607 | 5.41 | 9.51 (9.38-9.65) | *Reference* |  |
| Full sibling cohort |  |  |  |  |  |  |
| Exposed group | 783,781 | 55,040 | 7.06 | 9.69 (9.61-9.77) | 1.02 (1.01–1.04) |  |
| Unexposed group | 358,912 | 21,184 | 6.31 | 8.76 (8.64-8.87) | *Reference* |  |
| Exposure-discordant sibling pairs cohort | |  |  |  |  |  |
| Exposed group | 176,306 | 11,095 | 6.49 | 9.22 (9.05-9.40) | 0.91 (0.89–0.94) |  |
| Unexposed group | 176,306 | 11,433 | 6.51 | 9.36 (9.19-9.53) | *Reference* |  |

^a^ Baseline covariates were balanced in the singleton cohort by propensity score matching for the singleton cohort and were adjusted by multivariate Cox regression models in the full sibling cohort and exposure-discordant sibling pairs cohort.

### Table A4. Association of antibiotics exposure in 0-2 years old and subsequent autism spectrum disorder and/or attention-deficit/hyperactivity disorder, analyzed by separate outcomes

| Outcome | Singleton cohort (n) | Adjusted  hazard ratio^a^  (95% CI) | Exposure-discordant pairs sibling cohort (n) | Adjusted  hazard ratio^b^  (95% CI) |
| --- | --- | --- | --- | --- |
| ASD | 647,570 | 0.91, 0.88-0.95 | 352,612 | 0.88, 0.83-0.93 |
| ADHD | 647,570 | 1.08, 1.06-1.10 | 352,612 | 0.92, 0.90-0.94 |

ASD, autism spectrum disorder; ADHD, attention-deficit/hyperactivity disorder; aHR, adjusted hazard ratio; 95% CI, 95% confidence interval

^a^ The study adopts 1:1 greedy nearest neighbor propensity score matching without replacement, and the before-matching sample size is 946,581.

^b^ Baseline covariates were balanced in the singleton cohort by propensity score matching for the singleton cohort and were adjusted by multivariate Cox regression models in the full sibling cohort and exposure-discordant sibling pairs cohort.
